# Added Technology for Weight Management in Cardiac Rehabilitation: Biopsychosocial Factors and Rehabilitees’ Talk in a Mixed Method Study

**DOI:** 10.2196/78347

**Published:** 2026-03-02

**Authors:** Heli Lahtio, Teemu Paajanen, Hilkka Korpi, Ari Heinonen, Tuulikki Sjögren

**Affiliations:** 1 Faculty of Sport and Health Sciences University of Jyväskylä Jyväskylä Finland; 2 Physical Activity and Functional Capacity Research Group LAB University of Applied Sciences Lahti Finland; 3 Finnish Institute for Health and Welfare Helsinki Finland; 4 Welfare and Culture Oulu University of Applied Sciences Oulu Finland

**Keywords:** weight management, waist circumference, cardiac rehabilitation, remote technology, overweight, obesity

## Abstract

**Background:**

Hardly any previous studies have examined technology-based interventions among cardiac rehabilitees. The added value of technology and biopsychosocial factors in weight management should be studied more in cardiac rehabilitation.

**Objective:**

This mixed method study aimed to examine what biopsychosocial factors predict WC reduction and what factors arise from the talk of cardiac rehabilitees during 12-month cardiac rehabilitation.

**Methods:**

A total of 59 rehabilitees (mean age 60, SD 6 years; 48/59, 81.3% male) were randomly assigned in pairs into the experimental group (n=29) or the reference group (n=30). Biopsychosocial outcomes were measured at baseline and after 6 and 12 months with a 6-minute walk test (6MWT), the World Health Organization Quality of Life Brief Form questionnaire, body mass, BMI, WC, age, and sex. Rehabilitees’ experiences were investigated using focus group interviews. Both groups received conventional cardiac rehabilitation. In addition, the experimental group used technology. Multiple linear regression was used to predict WC change in 0-6 months and 0-12 months. The qualitative data were analyzed with thematic analysis. Integrative analysis was used to create a research-based model.

**Results:**

Group allocation predicted WC reduction (*P*=.007), with the experimental group showing a greater reduction (b=2.5 cm) than the reference group in the 0-6 month analysis. A significant interaction between the reference group and 6MWT was observed (*P*=.04), meaning that improvement in 6MWT was associated with WC reduction. Baseline WC was marginally associated with a greater reduction in WC (*P*=.05). None of the independent variables predicted WC reduction in 0- to 12-month analyses. Three themes arose from the interviews with the experimental group: meaningful factors related to weight management, a goal-oriented approach to weight loss, and monitoring body composition. Two themes emerged from the reference group: motivation for change and unstable weight management. According to the integrative analysis, belonging to the experimental group was important. The research-based model highlighted that the added value of remote technology on WC reduction was largest at the beginning of rehabilitation (0-6 months). The rehabilitees’ talk in the experimental group was goal-oriented, and they talked about the achieved changes. In the reference group, the talk remained motivational, and their body composition changes were unstable.

**Conclusions:**

The integrative model emphasizes the differences between the experimental and reference groups in weight management. The use of added remote technology seemed to explain the WC reduction. The rehabilitees reflected on behavioral changes in weight management. In the reference group, improved 6MWT distance was associated with decreased WC, but they talked about motivation for weight management and unstable changes in body composition. In the future, results should be verified by larger research groups and individual interviews, which can be combined with personal quantitative data.

**Trial Registration:**

ISRCTN Registry ISRCTN61225589; https://www.isrctn.com/ISRCTN61225589

## Introduction

### Background

Obesity is defined as a condition characterized by excess adiposity, with or without abnormal distribution or function of adipose tissue [1]. Obesity is not well understood. The causes are multifactorial, including genetic, metabolic, psychological, and environmental factors [1]. Obesity is a major risk factor of cardiovascular diseases (CVDs), with, for example, tobacco use, unhealthy diets, physical inactivity, and harmful use of alcohol [2]. Abdominal obesity with other risk factors, such as hypertension and diabetes, is associated with an increased risk of myocardial infarction [3]. Rehabilitees with obesity also experience events related to CVDs earlier, have longer years lived with CVD disability, and have shorter average life span [4].

CVDs are a leading cause of premature mortality worldwide. The incidence of CVDs has doubled between 1990 and 2019. Although CVDs can cause death, many rehabilitees continue to live with these conditions for many years [5]. The years lived with disability represent the number of years people live with a disease while experiencing a reduction in functional capacity. In 2019, the total years lived with disabilities attributed to CVDs amounted to 37 million years, reflecting a 106% increase from 1990 to 2019 [6].

Overweight or obesity is typically measured with BMI [1]. However, BMI does not differentiate fat and lean mass, reflect metabolic health, or consider body fat distribution [1]. Therefore, waist circumference (WC), or body fat percentage, might be more accurate for the evaluation of the perception of adiposity and obesity-related risks [1]. In every BMI category, the larger the WC, the higher the morbidity and mortality risk [1].

WC is a measurement to assess abdominal obesity [7,8]. It is a significant predictor of health [9] and cardiometabolic diseases [10]. High WC is a robust indicator of abdominal body fat, and thus is associated with CVDs [4], cardiovascular mortality [7], and recurrent atherosclerotic CVD, especially among men [11]. Increased WC is also associated with hypertension [9,12], diabetes mellitus, hypercholesterolemia, joint and low back pains, hyperuricemia, and obstructive sleep apnea syndrome [9]. Among people with CVDs, decreasing WC is an important target to prevent recurrence [11], morbidity and mortality [7], as well as decrease other well-known risk factors [7].

Weight loss is a complex and multidimensional issue, and therefore, obesity management needs comprehensive and multidimensional interventions [13]. Weight loss interventions should be individualized, multidisciplinary, and focused on realistic goals [14]. In the study by Rogerson et al [15], the rehabilitees experienced weight loss as an enduring and ongoing task. Weight loss involves physical, cognitive, behavioral, social, and environmental dimensions, which can, at the same time, either assist or inhibit weight loss. It has been stated that weight loss interventions concentrating only on the diet and increasing physical activity (PA) usually fail to achieve significant weight loss [16]. An individually designed diet, PA, and behavioral lifestyle interventions should be implemented to improve outcomes and adherence to weight loss interventions [17]. Psychological factors, such as motivation and self-efficacy, as well as behavioral factors, such as eating behavior, can affect the success of weight loss [18].

Technology-based weight loss interventions among rehabilitees with obesity have been studied widely [19-21], but the number of studies concerning technology-based weight loss interventions among cardiac rehabilitees is limited. However, single studies have been implemented. For example, den Uijl et al [22] found that the rehabilitees in cardiac rehabilitation involving activity trackers and behavioral change techniques lost significantly more weight than rehabilitees in standard cardiac rehabilitation. Technology-based interventions have been studied in preventing CVDs [23-25], in improving risk factors of CVDs [26], and in self-management of CVDs [27]. Positive results have been obtained in reducing CVD outcomes [25] and risk factors [24,26], as well as in the prevention and management of CVDs [23]. In health care, the role of artificial intelligence (AI) is increasing, for example, in medical image analysis, clinical decision support, and analyzing data from technological devices such as smartwatches [28]. In cardiac rehabilitation, AI may support the individuality, engagement, motivation, and self-direction of a cardiac rehabilitee [29,30]. In addition, AI-based interventions may be used in predicting CVDs, which enhance preventive strategies [31].

A systematic review, meta-analysis, and meta-regression study [32] found that web- and mobile-based interventions reduced BMI, WC, and body fat percentage. However, the interventions of the included studies were heterogeneous, and it was difficult to identify the real effects of technology-based interventions on body composition. Only 2 studies of 30 included cardiac rehabilitees, and 2 studies involved rehabilitees with cardiovascular risk factors [32]. A cluster randomized trial (CRT) study among cardiac rehabilitees [33] found that remote technology (Fitbit Charge HR activity monitor and Movendos mCoach) brought added value to WC reduction compared with conventional identical multi-professional rehabilitation without remote technology, but the reasons for this difference are unknown. It is important to obtain diverse and comprehensive knowledge about weight loss interventions among cardiac rehabilitees and the factors influencing weight management in order to improve cardiac rehabilitation and the functional ability of cardiac rehabilitees in the future.

### Objective

This mixed method study aimed to examine what biopsychosocial factors predict WC reduction and what factors arise from the speech of cardiac rehabilitees during 12-month cardiac rehabilitation. Based on the quantitative and qualitative results, an integrative research-based model of biopsychosocial factors and themes for weight management is created.

## Methods

### Study Design

This study (registration number ISRCTN61225589) was a mixed method study with a CRT and a qualitative study. The study design followed the convergent design, where the quantitative and qualitative data were collected at the same time, independently analyzed, and finally merged to combine the results [34].

The duration of the cardiac rehabilitation, implemented in a Finnish rehabilitation center, was 12 months in total. The measurements and interviews were carried out during in-rehabilitation periods at baseline, at 6 months, and at 12 months.

### Recruitment of Rehabilitees

A total of 59 rehabilitees were recruited from the rehabilitees attending a coronary heart disease rehabilitation course between September 2015 and May 2017. Participation in a course, which was funded by the Social Insurance Institution of Finland [35], required a physician’s referral. In Finland, rehabilitation courses for various diseases are available to all individuals diagnosed with the condition upon a physician’s referral. The rehabilitation courses are nationwide, not limited to a single well-being services county. Generally, rehabilitees can choose the rehabilitation center whose courses they want to participate in. In this study, all applicants to a specific rehabilitation center who met the inclusion criteria were enrolled as participants. The eligibility criteria for cardiac rehabilitation in Finland are being older than 18 years, a doctor’s statement recommending rehabilitation, and a diagnosed cardiac disease based on an appropriate diagnostic assessment. Further requirements include the following: the rehabilitee has limitations in work, study, or functional capacity caused by the disease, which impairs the ability to manage in everyday environments. The rehabilitee needs to have sufficient independent functional capacity to move in the rehabilitation environment. In addition, the rehabilitee’s initial treatment period has been completed, and the condition is stabilized [34].

The eligibility criteria for this study were being older than 18 years, having a diagnosed coronary heart disease, and independent basic-level management of IT and remote technology applications. To ensure the capability of using technology, the exclusion criteria included serious musculoskeletal disorders, cognitive diseases, and memory diseases that affect essential functional abilities. All rehabilitees fulfilled the inclusion criteria.

The study was conducted in a Finnish Rehabilitation Center, where rehabilitees came from all over Finland. The researchers divided an incoming group into 6 groups. The 2 types of groups were a conventional cardiac rehabilitation group with added remote technology (experimental) and a conventional cardiac rehabilitation group (reference) without added remote technology. The technologies used in this study were Movendos mCoach internet software (Movendos Ltd) and Fitbit Charge HR (Fitbit) accelerometers. The remote technology intervention clusters and reference clusters started in autumn (September to November), winter (December to February), and spring (March to May). The study design has been described in more detail in previously published articles [33,36,37].

### Rehabilitees

The mean age of the rehabilitees (n=59) was 60 (SD 6, range 41-66) years. A total of 77% (n=23) of the experimental group, and 86% (n=25) of the reference group were male. The rehabilitees had undergone a coronary angioplasty or coronary artery bypass 3-12 months before the study. At baseline, there were no statistically significant differences in the demographic and clinical characteristics between the experimental and reference groups.

### Randomization

Randomization was accomplished within the rehabilitation groups with sealed envelopes. A researcher from the gerontological center of the University of Jyväskylä, who was outside the research group, accomplished the randomization 3 times for 2 consecutive groups, under the supervision of 2 researchers (TS, Heikki Kivistö). A total of 59 rehabilitees were randomly assigned in pairs into (1) the remote technology intervention group (experimental group; n=29) or (2) the reference group (n=30). At the beginning of the rehabilitation, the researchers (TS and Heikki Kivistö) informed the rehabilitees about their rehabilitation group. The rehabilitation was implemented in the rehabilitees’ natural rehabilitation environment; it was not possible to blind the rehabilitees and caregivers due to the nature of the rehabilitation intervention and for ethical reasons. The outcome assessors were not blinded to the intervention, but the same educated persons who were not part of the research group performed the anthropometric measurements (a nurse specialized in heart diseases) and the 6-minute walk test (6MWT; a physiotherapist, specialized in heart diseases). The assessors came from outside the research group. The statistician (TP) was blinded.

### Ethical Considerations

This study was approved by the Ethics Committee of the Central Finland Health Care District (Dnro: 12 U/2015) on October 15, 2015. After randomization, the rehabilitees signed a written consent form about their participation in the study at the rehabilitation center. Participating in the study was voluntary, and rehabilitees were allowed to withdraw from the rehabilitation program at any time. All collected data were anonymized to ensure participant privacy and confidentiality, and all identifiable information was removed from the data. The rehabilitees did not receive any compensation for their involvement in the study. In Finland, rehabilitation is free for the rehabilitees, including free accommodation, program activities, and meals. In addition, rehabilitees who are employed receive a rehabilitation allowance for the loss of income due to the absence from work.

### Intervention

The rehabilitation courses aimed to promote the rehabilitees’ biopsychosocial functional ability and work ability [35]. Evidence-based rehabilitation methods and the Finnish Current Care Guidelines [38] were applied. Rehabilitation consisted of three 5-day in-rehabilitation periods in the rehabilitation center. Between these in-rehabilitation periods, the rehabilitees had two 6-month self-rehabilitation periods, when they followed home exercise programs in their own living environment.

Both the experimental and reference groups received conventional cardiac rehabilitation for 12 months, which included, for example, multidisciplinary rehabilitation, medical examination, physiotherapy, aqua therapy, gym training, and group discussions with a nutritionist, social worker, physiotherapist, psychologist, and physician. The rehabilitees received multi-professional information and pamphlets concerning, for example, CVDs and the management of daily activities, such as diets, PA, social security benefits, self-care, and self-rehabilitation while living with CVDs. From the experimental group rehabilitees, the researcher (Heikki Kivistö) collected individual and group-based PA information, stored in the database of Fitbit Charge HR (Fitbit) accelerometers, and sent the data to the physiotherapists. Based on that information, the rehabilitees received feedback on PA during the in-rehabilitation periods. The accelerometers were designed to be worn daily. After the intervention, all rehabilitees received personal information on PA and other biopsychosocial outcomes in paper reports. The only difference being the use of technology, the studied periods were equal in the experimental and reference groups, and the researchers (TS, Heikki Kivistö) from the University of Jyväskylä were in equal contact by phone with all rehabilitees during the different phases of the study.

The only difference between the reference and experimental groups was the use of technology. The reference groups did not have any technical devices, but they used printed materials and self-monitoring with paper and pen. The experimental groups used technological devices in monitoring their progress, communicating with the instructors, goal setting, self-monitoring, and giving feedback. The interventions have been described in more detail in our previous article [33].

### Outcome Measurements

This study used both the primary and secondary outcomes [33,36] of the study project. The primary outcome was a PA assessment [36], and the secondary outcomes were based on the biopsychosocial model: 6MWT, the World Health Organization Quality of Life Brief Form (WHOQOL-BREF) questionnaire, weight, BMI, WC, age, and gender. Additional secondary outcomes included the rehabilitees’ experiences collected with interviews. The outcome measurements were collected at baseline and after 6 and 12 months in the rehabilitation center.

6MWT is a reliable and valid field test [39] in which a rehabilitee walks at a self-selected pace as long as possible on a flat surface for a 6-minute period [40]. The heart rate, distance walked, and the symptoms were recorded. Anthropometric measurements included body mass, BMI, and WC. Body mass was measured by using a calibrated floor scale. The rehabilitees wore light clothes, such as T-shirts and shorts or tights, and they were without shoes. BMI was determined by dividing body mass by the height square in meters [41], and WC was measured on bare skin at the midpoint between the lowest rib and the iliac crest [7,42]. The WHOQOL-BREF questionnaire was used to measure quality of life. The questionnaire was performed on a paper format, and the researchers were responsible for completing it. The WHOQOL-BREF contains 26 questions. One question is related to overall health, and another question to overall quality of life. The other 24 questions are divided into 4 domains: physical (7 items), psychological (6 items), social (3 items), and environmental (8 items). Higher scores reflect a higher quality of life [43].

### Interviews

Semistructured focus group interviews were performed in the rehabilitation center at every follow-up point. The interview themes were formed by a group of researchers (TS, Arja Piirainen), and the interviews were conducted by one researcher (Heikki Kivistö). The interviews at the 6-month follow-up point were themed around experiences of rehabilitation and technology in the rehabilitee’s ordinary life and changes related to lifestyle. Additional themes concerned the personalization of rehabilitation and cooperation during rehabilitation. At the 12-month follow-up point, in addition to the previous themes, the interviews inquired about remote guidance and feedback, and meaningful experiences during rehabilitation. The full interview guides are presented in Multimedia Appendix 1. The interviews were implemented on the principle of an open interview, considering the themes. The interviews were recorded, and the recorded data were transcribed. Altogether, this study examined 312 pages of transcribed data, written in font size 12 and line spacing 1.0, including 4 pages of discussion about weight management in the experimental and reference groups. Saturation was not fully achieved because this study included rehabilitees from the rehabilitation course, where the interviews were part of the rehabilitation program. The schedule for all appointments was planned by the Social Insurance Institute of Finland, and the researchers had no possibility to extend the interviews. However, the rehabilitees in all target groups discussed the same themes, and no new themes emerged. In addition, several rehabilitees mentioned at the end of the focus groups that they had nothing further to add.

At the 6-month follow-up point, altogether, 47 rehabilitees participated (38 men and 9 women), and at the 12-month follow-up point, 30 rehabilitees (21 men and 9 women) participated in the interviews. Table 1 presents the number of rehabilitees in the focus groups.

**Table 1:** Group interviews with cardiac rehabilitees at 6 and 12 months follow-up points during their rehabilitation program.

	6 months		12 months	
	Men, n	Women, n	Men, n	Women, n
Group 1	7	1	2	1
Group 2	5	3	5	3
Group 3	7	2	7	2
Group 4	8	0	2	0
Group 5	7	1	3	2
Group 6	4	2	2	1
Total	38	9	21	9

### Quantitative, Qualitative, and Integrative Analyses

#### Statistical Analysis

Multiple linear regression was the primary method used for quantitative analysis. Before performing that, the Shapiro-Wilk test was performed to ensure the normality of the data. Multicollinearity was tested with a variance-inflating factor. After these tests, 2 separate dependent variables were analyzed using multiple linear regression: WC change from 0 to 6 months and from 0 to 12 months.

Multiple linear regression was performed with R Studio software (version 2024.12.0+467). First, all independent variables were tested with a dependent variable (WC change). The independent variable with the highest *P* value was iteratively removed in a stepwise backward elimination process. Finally, all statistically significant independent variables were included in the final model. If *P*<.05, predictors were considered statistically significant. Separate regression models were constructed for the 0-6-months and 0-12-months. Positive values indicate reductions, as the change variables (0-6 months and 0-12 months) were computed by subtracting the follow-up measurement from the baseline, meaning that baseline values were higher than at follow-up.

The interaction between the experimental group and 6MWT and the interaction between the reference group and 6MWT were tested in both models. The interaction was included in the model because, in the analysis of individual factors (0-6 months), it seemed significant and interesting. In addition, previous studies have found a negative correlation between 6MWT and body weight or BMI [44,45]. However, previous studies have not examined the interaction between factors related to weight loss and biopsychosocial factors, although the interaction seems statistically and clinically relevant from the point of view of the complex topic.

#### Qualitative Analysis

Thematic analysis of the qualitative data aimed to discover what the rehabilitees talk about their weight management. According to Braun and Clarke [46], one option for thematic analysis is to provide a detailed and nuanced account of one theme, or a group of themes within the data. Typically, this is related to a specific research question or area of interest. This thematic analysis focused on weight management themes, was inductive, and strongly linked to data [46].

Thematic analysis comprises six phases: (1) familiarizing with the data, (2) generating initial codes, (3) searching for themes, (4) reviewing themes, (5) defining and naming themes, and (6) producing the report [46,47]. In this study, researcher triangulation (HL, HK, TS) was used in every phase of the thematic analysis to ensure the quality of the qualitative analysis. In the first phase, the researchers became familiar with the data by reading it carefully and repeatedly. In the second phase, the data was coded. In the third phase, the codes were combined, compared, and analyzed. The analysis was inductive, and the themes were formed from the data (Table 2). In the fourth phase, the themes were processed by reviewing the coded data and by deciding whether the codes formed a coherent pattern or whether they would need reworking. After that, the researchers evaluated the themes in relation to the entire data set and decided whether the themes fit into the thematic map reflecting the meaning of the data. In this phase, the researchers reread the data to examine the themes and recoded the data arising from the newly created themes. In the fifth phase, the researchers created a definition and description of each theme, and in the final phase, they wrote the final analysis and description of the findings. At the initial phase, AI was used for language proofreading.

**Table 2:** An example of generating themes in qualitative analysis.

Quotes	Coding	Subthemes	Themes	Group and pages
“I’ve even gotten my wife exercise quite well.”	Has exercised with wife...	Weight management together	The meaningful factors related to weight management	G3, p. 15
“She’s lost more weight than me.”	...and wife has lost more weight than husband	Weight management together	The meaningful factors related to weight management	G3, p. 15
“It would be a personal (diet). So it would support everyone’s exercise, and generally help with losing weight as well.”	Personal diet in supporting exercising and weight loss	Personal diet in supporting exercise, weight loss and rehabilitation	The meaningful factors related to weight management	G3, p. 17
“But just that it would be personal and concrete – that would support this thing much more strongly.”	Personal diet in supporting the rehabilitation	Personal diet in supporting exercise, weight loss and rehabilitation	The meaningful factors related to weight management	G3, p. 17
“Five days, what you get here. It’s kind of... Should be more like rehabilitation, or kind of exercise.”	There should be more physical activity in the rehabilitation	Increasing the amount of physical activity in rehabilitation to prevent weight gain caused by prolonged sitting	The meaningful factors related to weight management	G5, p. 19
“Well, you could send a message there, like why people are gaining weight. It’s because we sit too much here. Yeah. We should just exercise more, and then...”	Sitting in the rehabilitation increases weight	Increasing the amount of physical activity in rehabilitation to prevent weight gain caused by prolonged sitting	The meaningful factors related to weight management	G5, p. 19

#### Integrative Analysis

In the integrative analysis, the main results from the quantitative and qualitative analyses were integrated. The integrative analysis was made following the steps of Skamagki et al [34]. Joint display was used in the first phase to organize the integration of quantitative and qualitative data. In the second phase, the linkages between quantitative and qualitative findings were defined. In the third phase, establishing relationships, the researchers looked for inconsistencies, alignments, or conflicting findings in the relationships between quantitative and qualitative data [34]. The fourth phase included interpreting and reporting the results.

## Results

### Overview

The results are presented in three phases: (1) quantitative, (2) qualitative, and (3) integrative study results. The results consider the outcomes from the experimental and the reference group, as well as the differences between them identified by both analysis methods. The results were used to develop a research-based model related to weight management and cardiac rehabilitation.

### Biopsychosocial Factors Predicting WC Reduction

In multiple linear regression, group allocation to the experimental and the reference groups significantly predicted WC reduction (*P*=.007), with the experimental group showing a greater reduction (b=2.5 cm) compared with the reference group. A significant interaction between the reference group and 6MWT was observed (*P*=.04): in the reference group, greater improvement in 6MWT was associated with a greater reduction in WC. No such association was observed in the experimental group (Table 2).

In addition, baseline WC was marginally associated with a greater reduction of WC (*P*=.05) in the 0- to 6-month analysis. According to the model, each 1 cm higher baseline WC was associated with a 0.07 cm greater reduction in WC.

None of the independent variables significantly predicted WC reduction in the 0-12 month analyses. For comparison, Table 3 also presents the 0- to 12-month results using the same model that was found to be optimal in the 0- to 6-month analysis (Tables 3 and 4).

**Table 3:** Results of multiple linear regression on waist circumference (WC) at 0-6 and 0-12 months.

Predictor	Estimate (0-6 months)	*t* test (*df*)	*P* value	Estimate (0-12 months)	*t* test (*df*)	*P* value
Experimental group (vs reference group)	2.52	2.83 (42)	.007	2.88	1.35 (21)	.18
6MWT^a^	–0.02	–1.91 (42)	.06	–0.06	–1.55 (21)	.14
WC at baseline (cm)	0.07	1.98 (42)	.05	0.06	1.38 (21)	.18
Experimental * 6MWT^b^	0.04	2.16 (42)	.04	0.05	1.29 (21)	.21

^a^6MWT: 6-minute walk test.

^b^Experimental * 6MWT is an interaction term between the intervention and 6MWT.

**Table 4:** Regression model fit and predictive accuracy statistics.

Metric	0-6 months	0-12 months
*R*²	0.23	0.19
Adjusted *R*^2^	0.16	0.03
*F* test (*df*)	3.16 (4, 42)	1.22 (4, 21)
*P* value	<.02	.33
Residual standard error	2.64	2.91

### Themes About Weight Management From the Talk of the Experimental Group

Three themes arose from the talk of the rehabilitees in the experimental group: the meaningful factors related to weight management, the goal-oriented approach to weight loss, and the monitoring of body composition. The group numbers and intervention follow-up points are presented in brackets after the quotations.

The talk about the meaningful factors related to weight management included the following subthemes: weight management together, personal diet instructions, and increasing PA in rehabilitation. The rehabilitees hoped for a personal diet which would support their weight loss, rehabilitation, and PA, as stated in the following example from a group interview:

It would be personalized (diet). So, it would still support everyone's physical activity and. And in general, support weight loss.

Group 3; 6 months

In addition, the rehabilitees talked about the amount of sitting during rehabilitation, resulting in a weight increase. Therefore, the rehabilitees hoped for more PA during the rehabilitation, as in the following example:

Well, you could send a message there asking why the fat keeps increasing. Because we sit here too much when we should... yeah... we should just move more, and then...

Group 5; 12 months

The goal-oriented approach to weight loss theme included the following subthemes: weight loss goals during rehabilitation, benefits of weight loss in lowering blood pressure, and the positive changes in muscle mass and body fat percentage. The rehabilitees mentioned that many rehabilitees aim for weight loss during rehabilitation, as in the following example:

Especially the goals—probably many have set a goal like 'I want to lose this much weight.

Group 3; 6 months

In addition, the rehabilitees remarked that as a result of weight loss, blood pressure decreases. This is illustrated by the following quote:

And of course, when the weight drops, it lowers blood pressure and everything else, and...

Group 3; 6 months

The monitoring body composition theme included 5 different perspectives. First, the rehabilitees mentioned that the weight remained the same because muscle weighs more than fat. Second, they discussed the results of their earlier body composition measurements. Third, they expressed a wish that measuring body composition would be included in the rehabilitation program. Fourth, they talked about their doubts about the reliability of the body composition measurement, and fifth, the positive changes in their body composition. This is illustrated by the following quote:

Why don't they measure at the beginning and the end of the Social Insurance Institute of Finland course? That would tell the truth, then.

Group 2; 12 months

### Themes About Weight Management From the Talk of the Reference Group

Two themes arose from the talk of the rehabilitees in the reference group: motivation for change and unstable weight management. The motivation for change theme comprised 2 subthemes: the motivating effect of the disease on weight management and lifestyle changes. The following quote illustrates how the rehabilitees talked about the effect of a disease on motivation:

Yeah, the motivation came from the heart attack, so it sort of started from there. It was a bit like my own desire to lose some weight and pay attention to what I'm eating and so on.

Group 4; 6 months

The rehabilitees mentioned motivating issues that affect rehabilitation, such as a healthy body, family, and the ability to work. This is illustrated by the following example:

Well, the motivation comes from wanting to get this, uh, body in shape so that I could be around as long as possible with my family and spend time with the kids, and, you know, do a bit of work on the side too.

Group 4; 6 months

The second theme, unstable weight management, contained contradictory discussions about weight change. Some rehabilitees achieved positive changes in their weight, while others did not report any changes in their weight, which disappointed them. An example is presented in the following quote:

I’ve lost a little weight and, and my endurance was better, and…

Group 1; 12 months

Some rehabilitees gained more weight during the rehabilitation, and they talked about the hope of losing the weight they gained. This is illustrated by the following examples:

Eating habits have changed, but the weight hasn’t gone down. That’s disappointing, for sure.

Group 1; 12 months

I've of course had a bit of a setback, I've gained a couple of kilos. But maybe it'll come off during the fall.

Group 1; 12 months

### Integrative Results of the Quantitative and Qualitative Data

**Figure 1:**
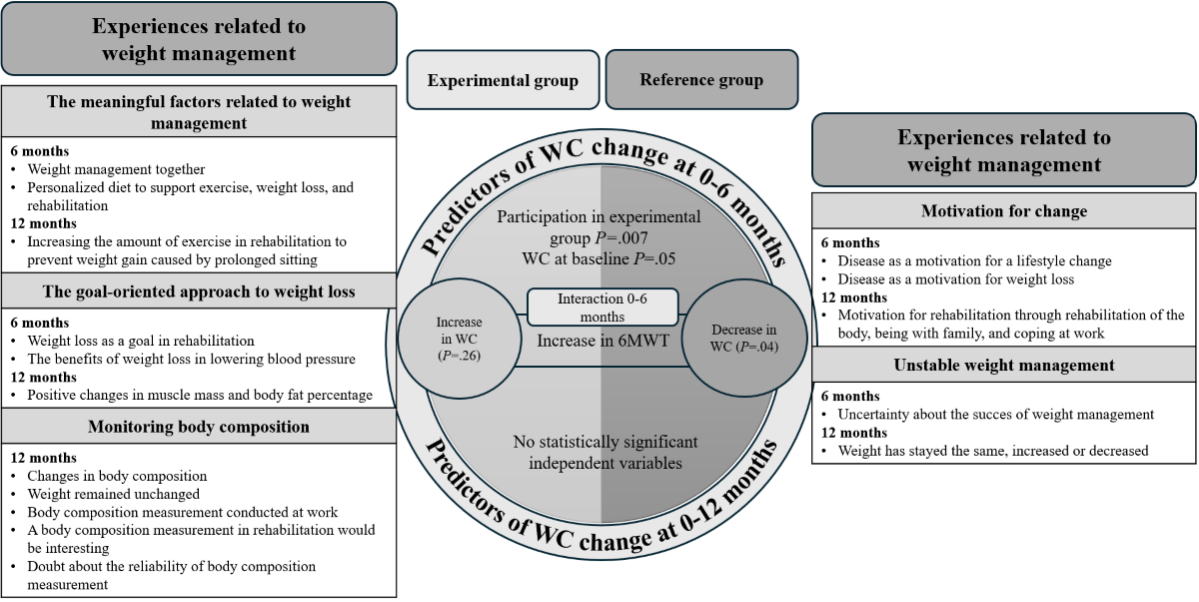
Integrative results with the experimental group on the left and the reference group on the right. 6MWT: 6-minute walk test; WC: waist circumference.

The integrative results of the quantitative and qualitative results are presented in Figure 1. The left side of the figure describes the results of the experimental group, and the right side describes the results of the reference group. The center of the figure describes the quantitative results at 0-6 and 0-12 month follow-up points. The sides of the figure describe the themes of the qualitative data.

The integrative model emphasizes that the experimental and reference groups differed in quantitative and qualitative results in terms of factors related to weight management. Whether the rehabilitee belonged to the experimental or the reference group was important in this study. The group was associated with WC reduction at 0-6 months (the center of Figure 1). The rehabilitees in the experimental group talked more about their weight management (left side of Figure 1) compared with the rehabilitees in the reference group (right side of Figure 1), and they seemed to have advanced in the process of change. The rehabilitees talked about elements of goal-oriented weight management, and they reflected on the changes they had achieved. In the reference group, improved 6MWT distance was associated with WC reduction at 0-6 months. In this group, the talk was more of a motivational speech, and the rehabilitees’ body composition changes were unstable. Furthermore, body composition changes arose from the talk of both groups. In the experimental group, the changes in weight management were positive, but in the reference group, the changes were unstable.

## Discussion

### Principal Findings

This mixed method study aimed to examine, during a 12-month cardiac rehabilitation, which biopsychosocial factors are related to WC reduction and what themes of weight management are meaningful for cardiac rehabilitees. A main finding was that allocation to the experimental group predicted greater WC reduction compared with the reference group. The integrative model indicated that the experimental and reference groups differed in the quantitative and qualitative results relating to weight management. Adding remote technology to conventional rehabilitation seemed to explain the WC reduction. The talk of rehabilitees was characterized by behavioral change and reflection on weight management. In the reference group, which did not use remote technology, an improved 6MWT distance in meters was associated with decreased WC. The rehabilitees’ talk dealt with motivation for weight management and unstable changes in body composition.

Our findings indicate that technology-based interventions are associated with WC reduction. However, other biopsychosocial variables were not associated with WC reduction. A similarity between the groups was found when a larger WC at baseline might predict significant WC reduction at 0-6 months (*P*=.05). This is an important finding as decreasing WC is essential in weight management because abdominal obesity is associated with CVDs [3]. This finding is in line with previous studies of the importance of baseline value to weight management changes during intervention [48-51]. Iwayama et al [48] found that a higher baseline WC was associated with a larger reduction of WC. Other studies have examined the association between early weight loss and long-term weight loss. Al-Abdullah et al [49] discovered that higher baseline weight and early weight loss were predictors of successful weight loss during an intervention. Höchsmann et al [50] and Farage et al [51] observed that initial weight loss predicted long-term weight loss. However, only one of these previous studies considered WC [48], and none of the studies considered cardiac rehabilitees.

This study found a statistically significant interaction between increased walking distance in 6MWT and decreased WC in the reference group, but not in the experimental group. The change in 6MWT was not statistically significant on its own, meaning that within the groups, the change in the walking distance was not associated with the WC change. One possible explanation for this finding is that the rehabilitees in the experimental group improved their nutrition or other factors that could not be captured with the quantitative measurements. Increased WC has been associated with decreased walking distance in 6MWT [52,53] among rehabilitees with rheumatoid arthrosis [52] and type 2 diabetes [53]. Among cardiac rehabilitees, Lenasi et al [54] found that age, WC, and New York Heart Association classification were predictors of 6MWT in rehabilitees with coronary artery disease. Similarly, Pepera et al [55] found that BMI >25 kg/m^2^ and age were independent predictors of poor 6MWT performance in rehabilitees with chronic heart failure. However, Sharma et al [56] studied healthy individuals and discovered a relation between 6MWT with BMI and waist-to-hip ratio. They found that when BMI increased, the 6MWT walking distance also increased. However, when the waist-to-hip ratio increased, the 6MWT walking distance decreased. Results regarding the interaction between 6MWT and other variables are contradictory, and more studies are needed to determine the relationship between 6MWT and weight management-related outcomes.

In this study, the significant predictors of WC change were statistically significant at the 0-6 month follow-up point, but none of the predictors were statistically significant at the 0-12 month follow-up point. Previous studies have found that the effects of interventions are initially more substantial but tend to diminish over the long term. In addition, typically engagement wanes over time as initial enthusiasm diminishes. Whiteley et al [57] found that during 6 months, engagement on technology-based weight loss intervention decreased 33%. In addition, Liljeroos et al [58] and Beleigoli et al [59] found that technology-based interventions were more effective in the short term, but the changes did not last in the long term.

The rehabilitees in the experimental group were more advanced in their weight management reflection compared with the rehabilitees in the reference group. The experimental group discussed the factors related to weight management, a goal-oriented approach to weight loss, and monitoring body composition. They talked about their body composition changes objectively, and their discussion was goal-oriented. The talk in the reference group was rather motivational, including motivation for change and unstable weight management. In line with the transtheoretical model, the rehabilitees in the reference group are in the preparation phase, in which the rehabilitees intend to take an action toward a change [60]. The rehabilitees in the experimental group are in the action phase. They have made changes, and their behavior change has been translated into action [61].

Previous studies have not compared the views of the rehabilitees in experimental and reference groups where the only difference was the use of technology. However, previous studies have examined the rehabilitees’ experiences of technology in weight management. Our results have similarities with previous studies. Lambert et al [62] found that an e-coach was beneficial in motivating the rehabilitees for a behavior change at the initial phase. However, the engagement diminished over time. Følling et al [63] found that activity monitors can strengthen or undermine the attempt at weight loss. Some rehabilitees mentioned that activity monitors motivated them to maintain PA and diet, providing proof of their effort. However, some rehabilitees felt that activity monitors highlighted their failure to change their lifestyle.

The rehabilitees in the experimental group discussed personalized diets in supporting weight loss, exercise, and rehabilitation. Previous studies and guidelines have stated that individually tailored weight loss programs can be important in weight loss [21,64,65]. In addition, the rehabilitees in this study mentioned the importance of social support in weight loss. Previous studies have found that social support from family and friends [57,59] as well as peer support is important in the weight loss process [62,66,67]. In addition, both groups talked about the effect of being overweight on diseases. The rehabilitees in the experimental group discussed the benefit of weight loss in lowering blood pressure, and the rehabilitees in the reference group discussed the motivation for weight loss arising from the cardiac disease. This finding is consistent with the study by Spreckley et al [68], who found that intrinsic motivators include the desire to improve health and the effort to improve weight-related conditions.

The experimental group talked about a goal-oriented approach to weight loss and weight loss as a goal in their rehabilitation. The rehabilitees in the reference group did not discuss their weight loss goals. The goal of weight loss might be imposed from outside, for example, by health care professionals, and people with obesity might assume that their goal should be weight loss. According to the study by Liu et al [69], external motivation is the key to initiating dietary changes. However, internal motivation is important for sustaining the behavioral change. This is in line with the self-determination theory, which suggests that intrinsic motivation, which is the most autonomous form of motivation, comes from a rehabilitee’s own interests, enjoyment, and satisfaction. Extrinsic motivation comes from outside (for example, from health care providers, social media, and society). When behavior is driven by external forces, long-term changes are typically unlike [70], whereas intrinsic goals result in improved self-regulation and long-term outcomes [71]. It might be important that the initial goal of weight loss comes, for example, from health care professionals, but for long-term changes, internal motivation is essential.

This study is the first mixed method study exploring technology-based weight loss intervention in cardiac rehabilitation. The study focuses on WC and incorporates several aspects that have not been studied before. For example, Dandeneau et al [72] performed a mixed method weight loss study on cardiac patients, but concentrated on adherence to the weight loss program as well as the strengths and limitations of the program. Liljeroos et al [58] conducted a mixed method study on the effects of a mHealth tool for rehabilitees with heart failure. However, they concentrated on the effectiveness and experiences of the mHealth tool with scales and questionnaires. Leung et al [73] examined, with mixed method study, the psychological factors of dietary and PA adherence in the early weight maintenance phase. However, they concentrated on participants with overweight and obesity, not cardiac rehabilitees.

In health care, obesity-related measurements are universal, and they categorize the rehabilitees to certain classes. This study also used commonly used measures for assessing weight loss. However, these measurements can be very embarrassing for the rehabilitees [74,75]. In behavior change interventions, these measurements are not necessarily optimal because of the stress and anxiety they may cause to the rehabilitees. According to Phelan et al [76], a welcoming and less threatening environment can be created by reducing focus on body weight and concentrating on obesity-related diseases and conditions, as well as encouraging behaviors that improve health and well-being. Anttila et al [37] formed 4 biopsychosocial profiles in technology-based cardiac intervention. The “feeling outsider” group had a high-risk behavior related to inactive behavior and being overweight. The “being uninterested” group had low PA levels and a poor self-assessed physiological quality of life. Both groups had large WC compared with the recommended value, and 6MWT was below the recommended value. The “reflecting benefit” group had high self-efficacy for exercise and an interest in health technology. The “enthusiastic users” group had high self-determination in exercise behavior but lacked self-efficacy in PA. The WC of the rehabilitees in these groups was closer to the recommended value, and their 6MWT was very close to the recommended value [37]. Individual measurements that do not necessarily concentrate on weight should be developed. This view has not been studied, so future studies should evaluate different measurements for overweight rehabilitees.

Because CVDs are a public health challenge and obesity is a risk factor for CVDs [2], weight management should be included in cardiac rehabilitation. However, it has not been a primary focus in there [77]. Technology may increase the engagement in cardiac rehabilitation [78] and lifestyle changes [79] as well as weight management [80]. Therefore, it should be considered to include technological components in cardiac rehabilitation and weight management. Health care systems are undergoing transformation by increasing the usability of technological devices. With technological devices, it is possible to achieve clinical and environmental benefits such as improving disease management, adherence, and access to health care. From an environmental view, digital health technologies decrease carbon emissions, waste generation, and energy consumption [81].

Our studies [32,33] produce gradually deepening information on the effectiveness of the added value of remote technology. Our previous systematic review with meta-analysis and meta-regression highlighted that previous RCT studies have compared highly heterogeneous experimental and control groups [32]. They have not compared experimental and control groups where the only difference was the use of technology. According to the findings of our CRT study [33], rehabilitation interventions using technology decreased WC more noticeably compared with the reference group without technology. The results from this study might indicate that technology brings added value to conventional rehabilitation and may support weight management, but it requires more studies in the future. To our best knowledge, only a few studies have compared experimental and control groups where the only difference is the technology use [25,82]. However, other studies have compared technology-based interventions to very heterogeneous control groups, which do not consider the added value of technology.

Future studies should examine the added value of technology by comparing interventions that are otherwise similar, but one group uses technology, and the other group does not. In addition, future studies should investigate the predictors of WC reduction and weight management, as well as weight management among cardiac rehabilitees.

In the future, AI is predicted to have a major role in smart health care. Smart health care offers several benefits by enhancing diagnostic accuracy, automating clinical workflows, and enabling personalized treatment strategies [28]. AI apps may support the individuality of cardiac rehabilitees’ rehabilitation, as well as their engagement, motivation, and self-direction [29,30]. In addition, AI-related solutions may enhance interaction between health care professionals and rehabilitees, support the monitoring of rehabilitation progress [29,30,83], and provide information on, for example, arrhythmias, heart rate variability, and PA [84,85]. However, research related to AI and rehabilitation is still largely limited to descriptive literature reviews [83-87] or studies conducted with non-experimental research designs [29,30,79]. At present, there is a substantial need for more precise evidence on effectiveness—obtained through randomized controlled trial designs—related to technology, including AI, and cardiac rehabilitation [88].

Future studies should investigate AI-based weight loss interventions among cardiac rehabilitees. With AI, it could be possible to, for example, personalize the interventions, provide real-time feedback, and improve adherence [28], which are key components in weight loss interventions. In cardiac rehabilitation, AI may be used in predicting CVDs [31], support early CVD detection, and enhance preventive strategies [31]. Furthermore, explainable AI may improve trust and accountability in health care, which are essential in technology-based interventions [89].

### Strengths and Limitations

This study has several strengths. First, it analyzed biopsychosocial factors broadly with multiple linear regressions and deepened the understanding of weight management with interviews. In addition, quantitative and qualitative results were combined in an integrative analysis. Second, the comprehensive cardiac rehabilitation intervention was implemented by a multiprofessional team. Third, the analyses and the randomization process were carefully implemented. The quantitative analyses were performed in collaboration with a statistician (TP), a principal investigator (TS), and a researcher (HL). The qualitative analyses used researcher triangulation throughout the process (HL, HK, and TS).

The strength of this study is that the analyses were discussed collaboratively by the entire research team, and the decisions were reached collectively. In the first linear regression analyses, BMI was included. However, BMI overwhelmed the effects of all other dependent variables. Therefore, BMI was excluded from linear regression. WC was considered more important than BMI, as previous studies have shown that in every BMI category, the larger the WC, the greater the risk of morbidity or mortality [1]. Therefore, WC was considered a more important factor than BMI in this study. One more strength of the study was its CRT design, which was integrated with a longitudinal interview component. To our knowledge, no similar mixed method studies have been conducted before.

A weakness of this study was the relatively small sample size. The protocol for cardiac rehabilitation, which is standardized by the Social Insurance Institute of Finland, and which is also responsible for rehabilitation costs, determined the sample size. The data collection took place over a 1-year period and included all cardiac rehabilitation groups participating in rehabilitation during that time. Due to the study schedule, it was not possible to collect a larger sample. Additionally, the group size was limited to a maximum of 10 rehabilitees. Therefore, the number of rehabilitees was limited. Because of the small sample size, care must be taken in generalizing the results beyond the target population. The rehabilitees in this study were residents across Finland, which increases the generalizability of this study in Finland. Globally, the results can be generalized in high-income countries. However, in low-income countries, for example, access to medical treatment is not necessarily possible. In addition, the number of cardiac rehabilitation studies is limited in low-income countries [90].

The characteristics of the rehabilitees in this study are similar to those in previous studies. For example, the mean age of the rehabilitees in this study was 60 years, and most of the rehabilitees were male. In previous studies, the mean age of the participants varied from 55 to 63 years [25,82,91-93]. In all these studies, most of the rehabilitees were male [25,82,91-93]. The higher proportion of men effects the generalizability of the results, as these results cannot be directly generalized to women. Fat distribution typically differs between women and men. In addition, biopsychosocial factors behind weight management may differ. For example, the prevalence of obesity is typically higher among women. However, men typically have more visceral fat deposits than women [94].

This study applied a qualitative thematic analysis to group interviews, which enabled the inclusion of a wider range of rehabilitees compared with individual interviews. However, focus group interviews may have affected the level of the rehabilitees’ talk by decreasing the discussion about weight management. A total of 312 pages of transcribed data were included in the qualitative study, but only 4 pages reflected weight management. Personal interviews would have been more reliable and made it possible to examine the experiences of the rehabilitees at an individual level. Despite the limited scope of discussion, several themes could be distinguished from the data.

The measurements used in this study are widely used but have some limitations. WC can vary across populations and between sexes. Another limitation is that WC does not reflect the accumulation of subcutaneous and visceral fat [1]. 6MWT and WHOQOL-BREF have limitations as well. Even though 6MWT is reliable and valid, small changes in the methodology of the test may affect the results [39]. The learning effect might be associated with improved walking distance. However, 6MWT has been validated for CVDs, and it can provide information about physiological reserve and prognosis [95]. WHOQOL-BREF is a reliable and valid questionnaire, but some studies have reported a ceiling effect [96-98]. However, the reliability of these measurements improved when the same nurse performed the body composition measurements at every measurement point, and one physiotherapist performed the measurements of 6MWT at every follow-up point.

### Conclusions

Both quantitative and qualitative results showed that technology-based intervention supported weight management. The integrative model emphasizes that the experimental and reference groups differed in quantitative and qualitative results in terms of factors related to weight management. The added use of remote technology to conventional rehabilitation seemed to explain the observed positive changes in WC. In addition, rehabilitees’ talk reflected behavioral changes in weight management, and the rehabilitees of the experimental group were further in their process of change when compared with the reference group. Technological devices may improve the engagement of cardiac rehabilitees in lifestyle changes and weight management and thus improve the well-being of the rehabilitees. In the future, the results should be verified for use by larger research groups, and interviews should be conducted as individual interviews that can be combined with personal quantitative data to achieve more personalized results. In addition, future studies should examine AI-based weight loss interventions for cardiac rehabilitees.

## Appendix

Multimedia Appendix 1 Focus group themes at 6- and 12-months follow-up points.

## Abbreviations

6MWT: 6-minute walk test
AI: artificial intelligence
CRT: cluster randomized trial
CVD: cardiovascular disease
PA: physical activity
WC: waist circumference
WHOQOL-BREF: World Health Organization Quality of Life Brief Form

## Data Availability

All data generated or analyzed during this study are included in this published article.
