# Supplementary material for: Added Technology for Weight Management in Cardiac Rehabilitation: Biopsychosocial Factors and Rehabilitees’ Talk in a Mixed Method Study
Source: J Med Internet Res. 2026 Mar 2;28:e78347. doi: 10.2196/78347 (PMC12993271; doi:10.2196/78347)
Supplement: Multimedia Appendix 1 [file jmir_v28i1e78347_app1.docx]

**The themes of focus group interviews at 6 months:**

Before the discussion, the rehabilitees will return the ‘Experiential Learning Self-Assessment Form’ (which will be given to them already on Monday). The themes to be addressed (1–2) will be discussed following the principles of an open interview. In each group, the discussion will start with these themes, and then the participants may continue the conversation freely.

1. **Experiences related to rehabilitation and the use of technology in everyday life and in lifestyle changes**
   1. Could you tell us how things have been going for you during these approximately six months since we last met?
      1. What kinds of changes have occurred in your everyday life during these six months?
      2. Which factors in your own life have influenced whether you have been able to make, or have not been able to make, changes related to your life?
         1. Factors related to oneself?
            1. What has been meaningful, own motivation

Self-monitoring of physical activity (e.g. Fitbit, Movendos)

- - - 1. Important people and factors related to one’s own life
         1. Family, work, hobbies, communities
      2. Factors related to the rehabilitation process
         1. Peers, professionals etc.

remote technology

- - - 1. Other things?

1. **Personalization of the rehabilitation process**
   1. How could rehabilitation (and the use of technology) better support your everyday life and/or own lifestyle changes?
      1. How should rehabilitation be developed so that it becomes more meaningful for you and so that you can commit to it better?
      2. How should rehabilitation be developed so that it better supports participation in rehabilitation and/or your activity (agency) in everyday life?
         1. During the in-rehabilitation period and between them
2. **Collaboration during the past 6 months**
   1. How have you experienced the collaboration during the past 6 months? (This theme should still be addressed if it has not come up in the earlier discussion.)

**The themes of focus group interviews at 12 months:**

The themes to be addressed (1–6) will be discussed following the principles of an open interview. In each group, the discussion will start with these themes, and then the participants may continue the conversation freely. Finally, a summary of the group’s results and a discussion of the results, possibly including their connection to the rehabilitation period.

1. **Experiences related to rehabilitation and the use of technology in everyday life and in lifestyle changes**
   1. Could you tell us how things have been going for you during these approximately six months since we last met?
      1. What kinds of changes have occurred in your everyday life during these six months?
      2. Which factors in your own life have influenced whether you have been able to make, or have not been able to make, changes related to your life?
         1. Factors related to oneself?
            1. What has been meaningful, own motivation

Self-monitoring of physical activity (e.g. Fitbit, Movendos)

- - - 1. Important people and factors related to one’s own life
         1. Family, work, hobbies, communities
      2. Factors related to the rehabilitation process
         1. Peers, professionals etc.

remote technology

- - - 1. What have you changed in your life?
      2. Other things?

1. **Personalization of the rehabilitation process**
   1. How could rehabilitation (and the use of technology) better support your everyday life and/or own lifestyle changes?
      1. Which experiences have been the most meaningful to you?
      2. How should rehabilitation be developed so that it becomes more meaningful for you and so that you can commit to it better?
      3. How should rehabilitation be developed so that it better supports participation in rehabilitation and/or your activity (agency) in everyday life?
         1. During the in-rehabilitation period and between them
2. **Collaboration during the past 6 months**
   1. How have you experienced the collaboration during the past 6 months? (This theme should still be addressed if it has not come up in the earlier discussion.)
3. **Remote guidance via computer**
   1. How have you experienced the guidance provided through the computer between rehabilitation periods?
   2. What has the staff’s presence been like during remote guidance?
   3. How would you like to develop remote guidance so that it meets your needs?
4. **Feedback on the group’s results during rehabilitation**
   1. How do you perceive the results?
   2. How do you think you will use them?
   3. How are they connected to the implementation of rehabilitation?
5. **Meaningful experiences during rehabilitation**
   1. What are the meaningful experiences at the beginning of rehabilitation,
   2. In the middle
   3. At the end?

Tuulikki Sjögren and Arja Piirainen
